# P-1122. Impact of an Antimicrobial and Diagnostic Stewardship Program in Children with Lower Respiratory Tract Infection admitted to Intensive Care Unit in Argentina

**DOI:** 10.1093/ofid/ofae631.1309

**Published:** 2025-01-29

**Authors:** Guadalupe Perez, Rodrigo Carmona, Marcela Zuazaga, Carolina Ansio, Ivana Kijko, Gabriela Gonzalez, Daniel Velardez, Adela Isasmendi, Agustina Tevez, Daniela Borgnia, Moira Taicz, Maria Teresa Rosanova, Rosa Bologna

**Affiliations:** Epidemiology and Infectious Diseases Department. Hospital de Pediatria Garrahan, Ciudad Autonoma de Buenos Aires, Argentina; Juan P Garrahan Hospital, Buenos Aires city, Ciudad Autonoma de Buenos Aires, Argentina; Juan P Garrahan Hospital, Buenos Aires city, Ciudad Autonoma de Buenos Aires, Argentina; Juan P Garrahan Hospital, Buenos Aires city, Ciudad Autonoma de Buenos Aires, Argentina; Juan P Garrahan Hospital, Buenos Aires city, Ciudad Autonoma de Buenos Aires, Argentina; Juan P Garrahan Hospital, Buenos Aires city, Ciudad Autonoma de Buenos Aires, Argentina; Juan P Garrahan Hospital, Buenos Aires city, Ciudad Autonoma de Buenos Aires, Argentina; Juan P Garrahan Hospital, Buenos Aires city, Ciudad Autonoma de Buenos Aires, Argentina; Juan P Garrahan Hospital, Buenos Aires city, Ciudad Autonoma de Buenos Aires, Argentina; Juan P Garrahan Hospital, Buenos Aires city, Ciudad Autonoma de Buenos Aires, Argentina; Juan P Garrahan Hospital, Buenos Aires city, Ciudad Autonoma de Buenos Aires, Argentina; Juan P Garrahan Hospital, Buenos Aires city, Ciudad Autonoma de Buenos Aires, Argentina; Juan P Garrahan Hospital, Buenos Aires city, Ciudad Autonoma de Buenos Aires, Argentina

## Abstract

**Background:**

Lower respiratory tract infection (LRTI) is a common cause of admission and antibiotic (ATB) overuse in children. Adequate use of diagnostic methods and ATB prescription in intensive care units (ICU) remain a challenge for antimicrobial stewardship programs.

**Aims:**

to evaluate the impact of a multilevel intervention to improve diagnostic testing and ATB use in children with LRTI admitted to ICU.

**Methods:**

Quasi-experimental, before-after study. Patients admitted to ICU from April to June 2023 (Pre Intervention period: PreI) and July to October 2023 (Post Intervention: PostI) with community acquired LRTI were included. Intervention consisted in educational activities, daily review and feedback of ATB prescription, development and dissemination of an institutional Guideline of ATB use in LTRI, including a Clinical Score to distinguish viral from bacterial pneumonia.

**Results:**

95 patients were included. Median age was 11 months (IQR 4-19).Fifty eight (61%) had any underlying condition, including: previous bronchiolitis N:16 (17%), Asthma N:8(8%) and Neurological Disorders N:7 (7%). Clinical diagnosis upon ICU admission was: bronchiolitis (N:42, 44%), asthmatic crisis (N: 33, 35%) and Pneumonia (N: 11, 12%). A viral etiology was documented in 76 patients (80%). The most common was RSV (N: 52, 55%) followed by Rhinovirus (N: 19, 20%). Viral coinfection was detected in 15 patients (16%). Sixty-three patients were admitted during PreI and 32 during PostI. Children admitted to ICU during PreI were more frequently healthy (PreI: 48% vs PostIn 22%, p 0.015), with bronchiolitis as admission diagnosis (PreI 56% vs PostI 23%, p 0.002) and confirmed respiratory infection (PreI 87% vs PostI 66%, p 0.01). In PreI more Blood cultures (PreI 61% vs PostI 51%, p 0.06) and tracheal aspirate cultures (PreI 48% vs PostI 22%, p 0.04) were performed. Empirical ATB were started on admission in 39 patients (62%) during PreI, and in 13 patients (41%) in PostI (p: 0.04). Adequate ATB prescription increased from 14% in the PreI to 37% in the PostI (p 0.01). Patients in PostI had shorter hospital length of stay (8 vs 14 days, p 0.02)

**Conclusion:**

A multilevel intervention in children with LRTI on admission to ICU improved the use of blood and tracheal aspirate cultures and ATB prescription. Length of stay decreased in PostI.

**Disclosures:**

**All Authors**: No reported disclosures

